# Silencing TUFM Inhibits Development of Monocrotaline-Induced Pulmonary Hypertension by Regulating Mitochondrial Autophagy via AMPK/mTOR Signal Pathway

**DOI:** 10.1155/2022/4931611

**Published:** 2022-07-27

**Authors:** Ruyuan Wei, Xin Lv, Changcun Fang, Chuanzhen Liu, Zengshan Ma, Kai Liu

**Affiliations:** ^1^Cheeloo College of Medicine, Shandong University, Jinan City, Shandong Province, 250012, China; ^2^Department of Cardiovascular Surgery, Thoracoscopy Institute of Cardiac Surgery, Qilu Hospital of Shandong University, Jinan City, Shandong Province, 250012, China

## Abstract

Pulmonary arterial hypertension (PAH) is an extremely malignant cardiovascular disease which mainly involves the uncontrollable proliferation of the pulmonary arterial smooth muscular cells (PASMCs). Recent studies have confirmed that mitochondria play an important role in the pathogenesis of pulmonary hypertension through sensing cell hypoxia, energy metabolism conversion, and apoptosis. As a mitochondrial membrane protein, TUFM has been regarded to be related to mitochondrial autophagy (mitophagy), apoptosis, and oxidative stress. Considering these factors are closely associated with the pathogenesis of PAH, we hypothesize that TUFM might play a role in the development of PAH. Our preliminary examination has showed TUFM mainly expressed in the PASMCs, and the subsequent test indicated an increased TUFM expression in the SMCs of pulmonary arteriole in monocrotaline- (MCT-) induced PAH rat model compared with the normal rat. The TUFM knockdown (Sh-TUFM) or overexpressed (OE-TUFM) rats were used to establish PAH by treating with MCT. A notable lower pulmonary arterial systolic pressure together with slightly morphological changes of pulmonary arteriole was observed in the Sh-TUFM group compared with the single MCT-induced PAH group. Increased levels of P62 and Bax and reduced LC3II/I, BECN1, and Bcl2 were detected in the Sh-TUFM group, while the expressions of these proteins in the OE-TUFM group were contrast to the results of the Sh-TUFM group. To elucidate the possible mechanism underlying biological effect of TUFM in PAH, PASMCs were treated with silence or overexpression of TUFM and then exposed to hypoxia condition. An obviously high levels of P62 and Bax along with a decreased LC3 II/I, BECN1, ULK1, Atg12, Atg13, and Bcl2 levels were noticed in cells with silence of TUFM. Moreover, the phosphorylated AMPK and mTOR which was well known in mitophagy modulating vary by the alternation of TUFM. These observations suggested that TUFM silence inhibits the development of MCT-induced PAH via AMPK/mTOR pathway.

## 1. Introduction

Pulmonary arterial hypertension (PAH) is an extremely malignant cardiovascular disease, characterized by the obstructive remodeling of the pulmonary vascular bed and progressive increased pulmonary vascular resistance, ultimately resulted in the functional decline of the right ventricle or right heart failure. Generally speaking, PAH is classified as idiopathic and secondary according to the different pathogenesis. However, the development of both types of PAH involves the excessive proliferation and the resistance to the apoptosis of the pulmonary arterial smooth muscle cells (SMCs). Even though the mechanism of PAH has been explored a lot with focusing on the pulmonary vascular SMCs, it still remains unclear due to the complexity and the interaction of the signal molecule in the regulatory pathway. Recent studies have confirmed that mitochondria play an important role in the pathogenesis of PAH by sensing hypoxia, oxidative stress, and apoptosis [1–3], which implies that the mitochondrial metabolism itself might be a pivotal regulator during the development of PAH.

Mitochondria determine the survival and death of cells: on the one hand, mitochondria are energy factory of cells, providing energy and biosynthesis substrates for cell survival through oxidative phosphorylation; on the other hand, abnormal accumulation of reactive oxygen species during mitochondrial metabolism can cause mitochondrial damage, leading to the release of proapoptotic proteins such as cytochrome C and other apoptotic factors [4, 5]. Mitochondrial autophagy or mitophagy as a necessary means of mitochondrial quality control selectively removes the damaged mitochondria debris, prevents the mitochondria from dysfunction, and guarantees the cells' normal function [6, 7]. It has been reported that the activation of mitophagy led to the over proliferation of the pulmonary arterial SMCs and promoted pulmonary vascular resistance [8]. However, the exact molecular mechanisms underlying mitophagy activation and PAH regulation remain poorly understood despite the identification of various mitophagy-associated proteins.

Mitochondrial translation elongation factor Tu (TUFM) locates in cytosol and partially on the outer membrane of mitochondria (OMM). It has been implicated in protein translation elongation and biosynthesis, oncogenesis, oxidative phosphorylation, and protein quality control [9, 10]. TUFM on the OMM modulates mitophagy by impeding Atg5-Atg12 formation and inhibits inflammation by binding to NLRX1 through its autophagic function [11, 12]. Alteration of TUFM expression has pleiotropic effects on the cellular networks and pathways and consequently impacts on cell physiology and homeostasis by causing imbalance in mitochondrial respiration chain and/or facilitates electron leakage, leading to the generation of ROS [13–15]. TUFM has been reported to be over expressed or decreased in several kinds of cancer invasion and metastasis by regarding as a chaperone-like proper ties in protein folding and renaturation under stress conditions [13, 16–18]. Since both the mitophagy and the cellular stress such as hypoxia and oxidative stress as well as inflammation are closely correlated to the pathogenesis of PAH in the literature [19, 20], TUFM is reasonably hypothesized to be a potentially central factor in PAH. Moreover, the most recent study has provided us with pulmonary arterial hypertension-related mitochondrial proteome analysis of the rat model, which the differential expression of TUFM was identified [21].

Nevertheless, although study on the conventional role of TUFM in peptide elongation and the clinical oncoproteomics were reported, little is known about the biological role of TUFM in the progression of PAH. Therefore, this study is aimed at presenting the TUFM expression profile in the PAH rat model, revealing the effect of TUFM on the PAH development in vivo, and exploring the possible regulatory mechanisms in vitro.

## 2. Materials and Methods

### 2.1. Animal Experiments

Animals received humane care, and the experiments were performed in accordance with the guidelines of Animal Care and Use Committee of Shandong University. Adult male Sprague–Dawley (SD) rats (200–220 g, 4–5 weeks old) were provided by Beijing Vital River Laboratory Animal Technology Co. Ltd. Monocrotaline (MCT, C2401, Sigma-Aldrich, USA) was injected into the subcutaneous tissue (60 mg/kg) to induce progressive pulmonary arterial hypertension at the end of 4 weeks according to the previous methods [22, 23]. In the preliminary experiment, normal control rats (*n* = 6) and MCT-induced PAH model (*n* = 6) were used to detect the differential expression of TUFM. In the following experiment, 36 rats were divided into 6 groups (*n* = 6 for each group): (1) control group (Ctrl), (2) MCT-induced PAH group (PAH), (3) TUFM overexpressed group (OE), (4) OE-negative control group (OE-NC, rats received OE empty vehicle), (5) Sh-TUFM group (Sh-TUFM were knockdown), and (6) Sh-negative control group (Sh-NC, rats were received Sh-TUFM empty vector). The control group was given the equal amount normal saline as placebo while for the other five groups, MCT was injected to establish PAH rats. Based on the NCBI-related sequence (NM_001106295.1) and according to the expression and distribution of TUFM in the previous experiments, AAV9 serotype adenoassociated virus with sm22a (Vigene Biosciences, USA) was selected as the SMC-specific promoter. For OE and Sh-TUFM groups, 1 ml vehicle contained 10^12^GC/ml titer of knockdown or overexpression was injected into the caudal vein of each rat in the respective group. Equal volume of corresponding empty vehicle was used as the negative control. The sequence was as follows: Sh-TUFM: sense, 5′-CCCUGGUCAUGCAGAUUAUTT-3′ and antisense, 5′-AUAAUCUGCAUGACCAGGGTT-3′ and Sh-NC: sense, 5′-UUCUCCGAACGUGUCACGUTT-3′ and antisense, 5′-ACGUGACACGUUCGGAGAATT-3′.

### 2.2. Hemodynamic Measurements and Tissue Collection

Animals were anesthetized and immobilized on the bench board. Pulsed Doppler (Vevo 2100; Visualsonics) was performed to detect the pulmonary artery Doppler signals, and the pulmonary artery acceleration time (PAAT) was measured. Each measurement was repeated for 3 times, and the mean value was calculated. The hemodynamic data were measured directly using a catheter. Animals were anesthetized and supported with respiratory machine; then, thoracotomy was performed. A 3F polyethylene catheter was inserted into the main pulmonary artery to measure the pulmonary artery pressure according to the previous method [24]. The catheters were linked to a transducer connecting with a multichannel physiologic monitoring recorder (Powerlab8/30; AD Instruments Pty Ltd.) for measurement and record of the pressure. The displayed digits were recorded for 3 times per animal, and the average pressure was regarded as the respective indicators.

The right lung and heart tissues were flushed with normal saline to clear blood and then frozen in liquid nitrogen in preparation for western blot analysis. The left lung was fixed with 4% paraformaldehyde, and paraffin section was made for hematoxylin and eosin (HE) stain and histological examination.

### 2.3. Morphology and Immunohistochemistry Analysis

The paraffin slice was stained with HE, and the arteriola in the lung were observed under optical microscope (Olympus BX51). The vessels between 50 *μ*m and 200 *μ*m in the external diameter (ED) were chosen randomly under low-power fields for the analysis of medial hypertrophy. ED and medial thickness (MT) were measured, and the remodeling of arteriola was calculated as percent MT (MT% = 2 × MT/ED × 100) as described previously [24, 25].

Paraffin section was treated with hydrogen peroxide, blocked with 5% bovine serum albumin, and then incubated with the primary antibodies (TUFM, 1 : 200, ab173300, Abcam; *α*-SMA (BOSTER, BM0002, 1 : 1000 dilution) and CD31, Abcam, ab222783, 1 : 2000 dilution) overnight at 4°C for immunohistochemistry or immunofluorescence test. After the uncombined primary antibody was washed, the slice was incubated with second antibody for 30 min at 37°C. The primary antibody was substituted by PBS for negative control. Peroxidase activity was visualized by a color reaction with diaminobenzidine, and a positive result was represented by brown color. The slices were counterstained with hematoxylin and mounted. For the immunofluorescence chemistry examination of the preliminary experiment, all the procedures were performed in a dark place and the slices were counterstained with DAPI to label the cell nucleus. The pathologist reviewing the slices was blind to the experimental design. The slice of immunohistochemistry and immunofluorescence were analyzed under the optical microscope or the fluorescence microscope.

### 2.4. Cell Culture and Gene Transfection

The human pulmonary arterial SMCs (PASMC) were incubated in normoxia condition (5% CO_2_ at 37°C) and hypoxia incubator (3% O_2_, 5% CO_2_, and 92% N_2_), respectively. Small inference RNA was designed for knockdown TUFM, and plasmid carried TUFM was transfected to overexpress TUFM. Generally, once adherent cells were at >80% confluence in the 6-well plates, the plasmid carried TUFM gene or empty plasmid is mixed with buffer, shaking for 1 minute, then mixed with jetprime transfection reagent (Polyplus, France), shaking for 10 minutes, and then added to the cells. After coincubated with the plasmid for 6 hours, the cell supernatant was changed to fresh medium. Thereafter, the protein was collected 48-72 hours later. Two sequences of SiRNA (GenePharma) were selected from the alternative options for this experiment. When the cell grows to about 30%-50%, the two interfering sequences and corresponding empty sequences are transfected to the cells. The other procedures were identical with the previous methods. The sequence is as follows: Si-TUFM-1: sense, 5′-CACCGAGUUUGGCUAUAAATT-3′ and antisense, 5′-UUUAUAGCCAAACUCGGUGTT-3′; Si-TUFM-2: sense, 5′-GGGCUAAGUUCAAGAAGUATT-3′ and antisense, 5′-UACUUCUUGAACUUAGCCCTT-3′; and Si-NC: sense, 5′-UUCUCCGAACGUGUCACGUTT-3′ and antisense, 5′-ACGUGACACGUUCGGAGAATT-3′.

First of all, TUFM test was performed in PASMCs cultured in normoxia (Ctrl group) or hypoxia condition (Hyp group) to found the expressions in different condition. At the meanwhile, empty plasmid or sequence was transfected to the cells in normoxia or hypoxia culture to exclude the interference of empty vehicle during the gene transfection. Then, in the subsequent examination, cells were treated with SiRNA-1, SiRNA-2, overexpression (OE), and the corresponding empty vehicle (Si-NC or OE-NC), respectively, before the different condition culture.

### 2.5. Western Blot

Protein was drawn from the collected SMCs or lung tissues on ice by treatment with lysis buffer, phenylmethanesulfonyl fluoride (PMSF), and phosphatase inhibitors (both from Beyotime). The protein supernatants were centrifuged at 4°C (12,000 rpm for 15 min) to remove the tissue fragments of the sediments. Concentrations of protein were determined by the Bio-Rad protein assay instrument, and the protein was boiled with loading buffer. Equal amounts of protein were added into the wells of gel electrophoresis and transferred onto polyvinylidene fluoride membrane. The membranes were blocked with 5% fat-free milk for 2 h and then incubated with the respective primary antibodies overnight at 4°C. The related primary antibody included TUFM (Abcam, ab173300, 1 : 10000 dilution), LC3 (CST, #3868, 1 : 1000 dilution), BECN1 (CST, #3495, 1 : 1000 dilution), P62 (CST, #39749, 1 : 1000 dilution), ATG13 (Proteintech, #18258-1-AP, 1 : 1000 dilution), *α*-SMA (BOSTER, BM0002, 1 : 1000 dilution), CD31 (Abcam, ab222783, 1 : 2000 dilution), Bax (Abcam, ab182734, 1 : 1000 dilution), Bcl2 (HUABIO, ET1702-53, 1 : 1000 dilution), AMPK (Bioss, bs-5551R, 1 : 1000 dilution), p-AMPK (Bioss, bs-2771R, 1 : 1000 dilution), mTOR (Bioss, bs-3494R, 1 : 1000 dilution), p-mTOR (Bioss, bs-3495R, 1 : 1000 dilution), ULK1 (BOSTER, A00584-1, 1 : 1000 dilution), Apaf-1 (HUABIO, ET1607-12, 1 : 1000 dilution), ATG12 (CST, #4180, 1 : 1000 dilution), ATG16L1 (CST, #8089, 1 : 1000 dilution), and *β*-actin (CST, #4970, 1 : 1000 dilution) which was used as an internal reference. The blots were incubated with secondary antibodies conjugated to horseradish peroxidase for 1 h at room temperature with continuous shaking. After washing, the protein blots were detected using an enhanced chemiluminescence kit (Millipore) and exposed to X-ray film. The recognized bands were quantified by Tanon 4800 (Alpha Innotech). Densitometry analysis was performed using the ImageJ software.

### 2.6. CCK-8 for the Cell Viability Assay

Cell viability was detected using a CCK-8 Kit (GLPBIO) according to the manufacturer's instructions. The absorbance was measured at 450 nm. Cells were treated with different conditions as mentioned above and then trypsinized and evenly seeded into 96-well plates. CCK-8 solvent of 10 *μ*l mixing with 90 *μ*l medium was added to the wells, and the cells were incubated for 2 hours at 37°C. Absorbance was measured at the 12, 24, and 36 h, respectively.

### 2.7. EdU for Cell Proliferation Assay

Cells were treated and incubated with medium in 96-well plates for 24 hours. Medium was sucked out, and the cells were washed with PBS three times; then, the prepared medium containing EdU reagent (RIBOBIO) was added. Cells were incubated for another 3 hours according to the manufacturer's instruction and stained with DAPI for the nuclear label. Laser confocal microscopy was adopted for observation of the labeled cells.

### 2.8. Transmission Electron Microscope

The cells were treated for 24 hours, fixed with glutaraldehyde, washed three times with PBS, then treated with 1% osmic acid, rinsed again with PBS, and dehydrated with propanol. The cells were embedded in epoxy resin and 2% uranium acetate-saturated alcohol solution. The autophagy bodies of different treatment groups were observed by transmission electron microscope (HITACHI, HT7700, Japan).

### 2.9. Statistical Analysis

SPSS 20.0 for windows was used for all statistical analysis, and figures were prepared using the GraphPad Prism 6.0 software. Variables were presented as mean ± standard deviation. For comparisons between 2 groups, Student *t*-test (unpaired and 2-tailed) was used. For comparisons between multiple groups, 1-way ANOVA followed by Bonferroni post hoc test was conducted for significance. All statistical tests used two-sided tests of significance. A value of *P* < 0.05 was considered statistically significant.

## 3. Results

### 3.1. Establishment of PAH Model Induced by MCT

**Figure 1:**
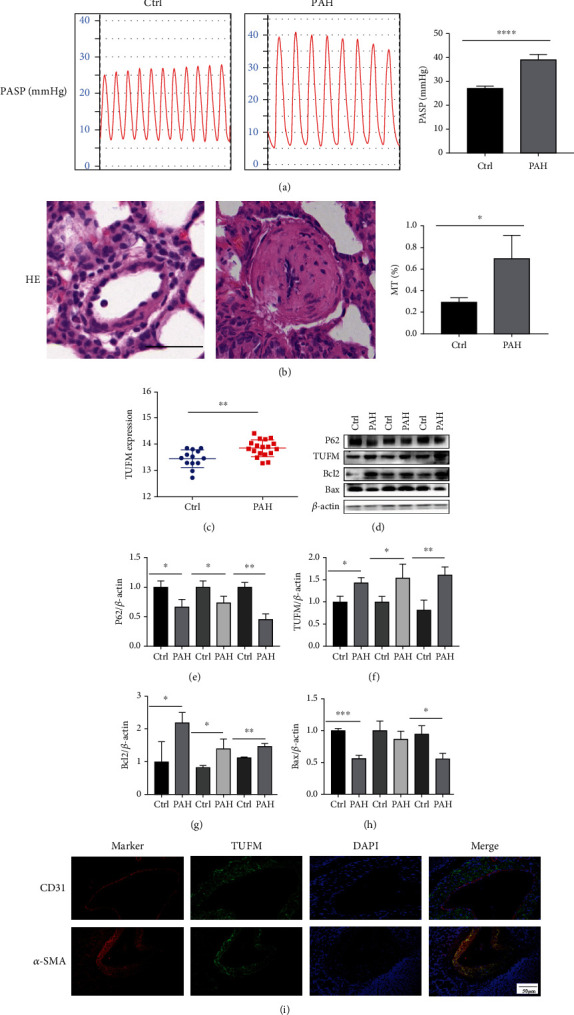
Monocrotaline-induced pulmonary hypertension and the expression of TUFM in the pulmonary hypertension and normal control rats. (a) 28 days after monocrotaline induction of the model, the pulmonary arterial systolic pressure measured using the system. (b) Hematoxylin and eosin (HE) staining for the pulmonary arterioles of normal control and monocrotaline-induced PAH model. Scale bar: 50 *μ*m. (c) Differential expression of TUFM from GSE15197 database analysis. (d) The initial test for TUFM, mitophagy marker P62, and apoptosis indicator Bax and Bcl2 expression in the normal control and monocrotaline-induced PAH rat. (e–h) Densitometry data represent the intensity of the quantitative expression of P62, TUFM, Bcl2, and Bax proteins. (i) Immunofluorescence chemistry examination indicated the location profile of TUFM in pulmonary arteries. Scale bar: 50 *μ*m. Ctrl: normal control; PAH: pulmonary arterial hypertension. Data is presented as mean ± SD. *n* = 6 each group. ^∗^*P* < 0.05 vs. Ctrl group, ^∗∗^*P* < 0.01, and ^∗∗∗^*P* < 0.001.

In the preliminary experiment, four weeks after MCT treatment, pulmonary arterial pressure was significantly increased in the PAH group (Figure 1(a)). The pulmonary vascular tunica media in the PAH group was thicker than the normal control group (Figure 1(b)), which indicated MCT-induced PAH model was successful.

### 3.2. High Expression of TUFM in MCT-Induced PAH

GSE15197 data were retrieved from the gene expression omnibus (GEO) and were analyzed. We found that TUFM was overexpressed in PAH compared with the normal control (Figure 1(c)). So, we tested the expression of TUFM in MCT-induced PAH rat model. The result was identified with the analysis of GSE15197 data that expression of TUFM was higher in MCT-induced PAH compared with the normal control group. As TUFM was involved in mitophagy and apoptosis, we also choose P62, Bcl2, and Bax as the corresponding factors in the initial test. The results showed a decreased P62, increased Bcl2, and reduced Bax level in the PAH group (Figures 1(d)–1(h)). Furthermore, we detected the location of TUFM in the small pulmonary artery. Immunofluorescence chemistry examination showed that TUFM mainly located in the SMCs of the pulmonary artery, while absent or weakly expressed in the endothelial cells (Figure 1(i)).

### 3.3. TUFM Knockdown Inhibits the Development of PAH

**Figure 2:**
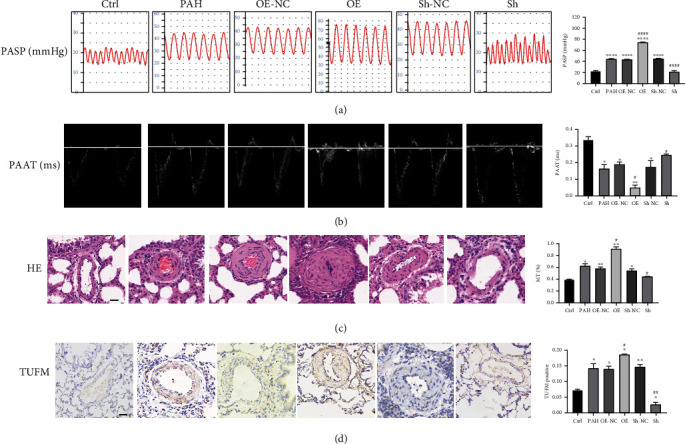
Characterization of different treated rats. (a) Images and ratio comparison of pulmonary arterial systolic pressure (mmHg). (b) Pulmonary artery accelerated time (ms). (c) Hematoxylin and eosin stain of paraffin slice. (d) Immunohistochemistry for TUFM in each group. Scale bar: 50 *μ*m; Ctrl: normal control; PAH: pulmonary arterial hypertension; OE-NC: overexpression negative control; OE: overexpression of TUFM; Sh-NC: Sh-TUFM negative control; Sh: Sh-TUFM group; PASP: pulmonary arterial systolic pressure; PAAT: pulmonary arterial accelerated time; HE: hematoxylin and eosin stain. Data are presented as mean ± SD. *n* = 6 each group. ^∗^*P* < 0.05 vs. Ctrl group, ^∗∗^*P* < 0.01, and ^∗∗∗^*P* < 0.001; ^#^*P* < 0.05 vs. PAH group and ^####^*P* < 0.0001.

To explore the role of TUFM in the development of PAH, Sh-TUFM and OE-TUFM rat received MCT to establish PAH for the further study. Four weeks of MCT treatment, the pulmonary arterial pressure was significantly increased in the OE group but only a slightly elevation was found in the Sh group (Figure 2(a)). In addition, the echocardiographic examination showed a relative longer time of PAAT in the Sh group but a shorter time in the OE group, which indicated that the absence of TUFM decelerated the PAAT and decreased the resistance of pulmonary arteries (Figure 2(b)) while overexpression of TUFM increased the pressure of pulmonary circulation. The morphology of pulmonary arteriole in the Sh or OE group was also in accordance with the changes of pulmonary arterial pressure: the lumens of pulmonary arteriole of the OE group were nearly obstructed while the vascular medial wall became mildly thick after MCT treatment in the Sh group (Figure 2(c)). The thickness of tunica media represented by MT% in the OE group was obviously higher than those in the other groups (Figure 2(c)).

### 3.4. Effect of TUFM on Mitophagy and Apoptosis in PAH

**Figure 3:**
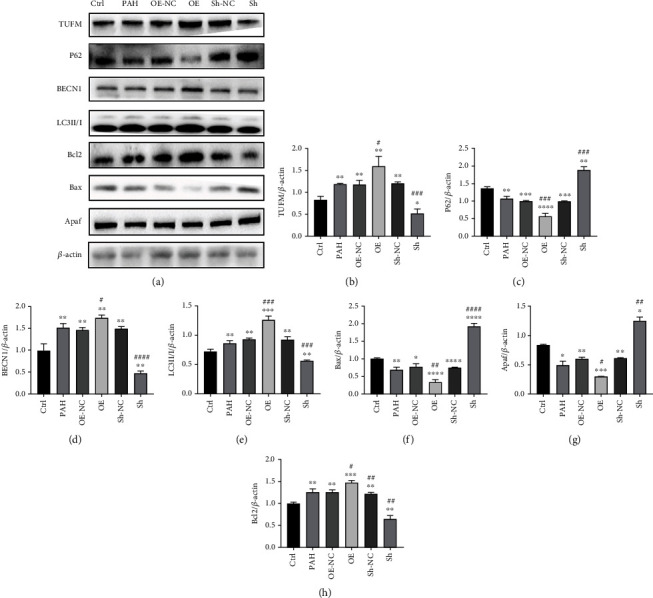
Expressions of TUFM and the autophagy indicators in different treated groups. (a) Western blot of TUFM, LC3II/I, Bax, Bcl2, BECN1, P62, and Apaf expression in each group as indicated. (b–h) Densitometry data represent the intensity of each group. Ctrl: normal control; PAH: pulmonary arterial hypertension; OE-NC: overexpression negative control; OE: overexpression of TUFM; Sh-NC: Sh-TUFM negative control; Sh: Sh-TUFM group. Data is presented as mean ± SD. *n* = 6 each group. ^∗^*P* < 0.05 vs. Ctrl group, ^∗∗^*P* < 0.01, ^∗∗∗^*P* < 0.001, and ^∗∗∗∗^*P* < 0.0001; ^#^*P* < 0.05 vs. PAH group, ^##^*P* < 0.01, ^###^*P* < 0.001, and ^####^*P* < 0.0001.

Consistent with the preliminary examinations, both immunohistochemistry analysis and the western blot showed an increased level of TUFM in PAH apart from the Sh group (Figure 2(d)). TUFM was highly expressed in PASMCs after treatment of MCT and nearly unexpressed in the normal control group (Figure 2(d)). During the development of PAH, knockdown of TUFM by ShRNA not only showed a decreased TUFM level but also demonstrated a reduced expression of mitophagy indicator, such as BECN1 and LC3II/I, along with an increased P62 level (Figures 3(a) and 3(b)–3(e)). Simultaneously, antiapoptosis factor Bcl2 was reduced, while the apoptosis activator Apaf and Bax raised under the condition of TUFM absence (Figures 3(a) and 3(f)–3(h)). In contrast, the mitophagy and apoptosis-related factors mentioned above in the OE group changed in an opposite direction of the Sh group (Figure 3). These evidences revealed that TUFM silence attenuated MCT-induced PAH by promoting apoptosis and relieved mitophagy.

### 3.5. Hypoxia Induced the Upregulation of TUFM in PASMC

**Figure 4:**
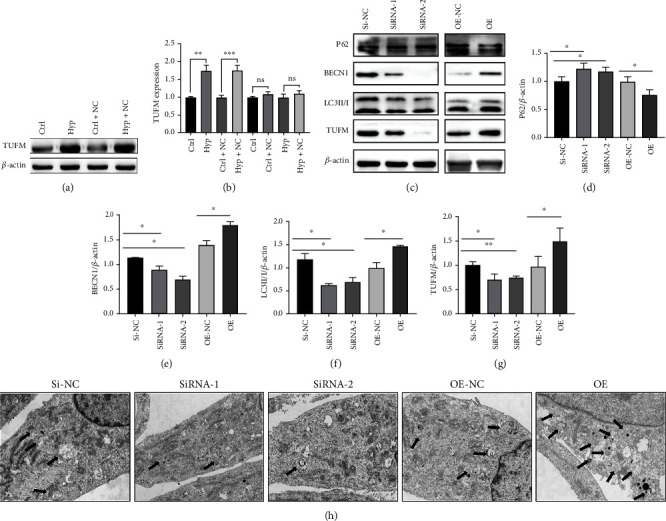
Hypoxia induced the overexpression of TUFM, and the silence of TUFM decreased the mitophagy of smooth muscle cells under hypoxia condition. (a) TUFM expression in smooth muscle cells under normoxia and hypoxia conditions. (b) Schematic representation of the quantitative expression of TUFM protein. (c) TUFM, LC3 II/I, BECN1, and P62 expression in hypoxia of each group. (d–g) Densitometry data represent the intensity of each group. (h) Autophagy of pulmonary artery smooth muscle cells collected by transmission electron microscopy in each group. Scale bar: 2 *μ*m. Ctrl: control; Hyp: hypoxia; Ctrl-NC: control+empty vehicle; Hyp-NC: hypoxia+empty vehicle; OE-NC: negative control of TUFM overexpression; OE: overexpression of TUFM; Si-NC: small inference RNA of TUFM negative control; SiRNA: small inference RNA of TUFM. Data is presented as mean ± SD, ^∗^*P* < 0.05, ^∗∗^*P* < 0.01, and ^∗∗∗^*P* < 0.001.

To determine the TUFM expression under a hypoxia condition, the protein level was measured by western blot. TUFM was upregulated by the stimulation of hypoxia while low level of TUFM in the normoxia condition cells. The empty vehicle has no influence on the TUFM expression of PASMC in both hypoxia and normoxia culture (Figures 4(a) and 4(b)). Next, cells were treated with small inference RNA or the TUFM overexpression plasmid first and then exposed to hypoxia condition in the following examinations.

### 3.6. TUFM Silence Alleviated the Mitophagy Induced by Hypoxia

In order to further explore the effect of TUFM on the mitophagy of PASMCs during the hypoxia culture, some mitophagy indicators were tested. Consistent with the in vivo results, silence of TUFM enhanced the expressions of the P62 and reduced BECN1 and LC3II/I levels. Contrasty to the phenomenon induced by Si-TUFM, lower level of P62 and high expression of BECN1 and LC3II/I were observed in the OE group, which represented an excessive mitophagy (Figures 4(c)–4(g)). These findings suggested that the absence of TUFM contributed to the attenuation of mitophagy in a hypoxia environment.

### 3.7. Effect of TUFM on the Morphology of PASMCs under Hypoxia Condition

Considering the role of TUFM in mitophagy, transmission electron microscopic examination was carried out to detect the morphology of cells. In the Si-NC and OE-NC groups, the mitochondria were slightly swollen but still with an intact membrane under hypoxia. The cristae decreased in partial damaged mitochondria, and the damaged mitochondria was packed in the autophagosome. Structure of the most mitochondria in Si-TUFM cells was integrated, the cristae were existed, and the membrane was intact. Autophagic lysosome was seldomly detected under the view of transmission electron microscopy. However, in the OE group, some mitochondrial membranes were damaged and disintegrated; the cristae were broken and disappeared. Some autophagosomes containing the damaged mitochondrial fragments were observed (Figure 4(h)).

### 3.8. Absence of TUFM Improved the Apoptosis of PASMCs under Hypoxia Condition

**Figure 5:**
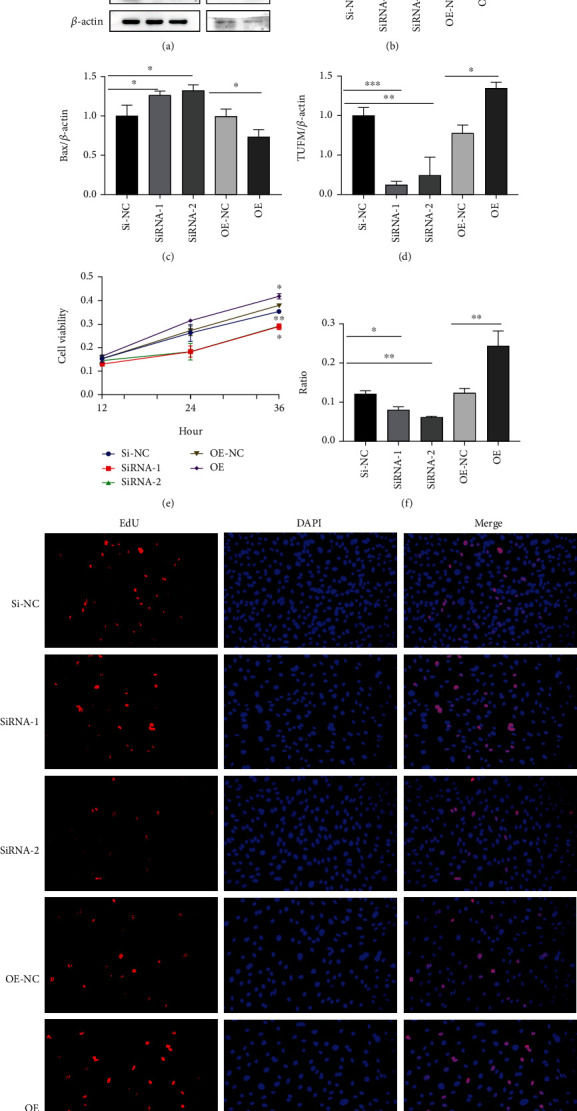
Absence of TUFM improved the apoptosis and reduced the proliferation of PASMCs. (a) TUFM, Bax, and Bcl2 expression under hypoxia condition. (b–d) Densitometry data represent the intensity of each group. (e) Cell viability tested by CCK-8 under hypoxia condition. (f) Quantification of proliferation level in smooth muscle cell by EdU assay kit. (g) Cell proliferation activity of each group under hypoxia. Scale bar: 50 *μ*m. Data is presented as mean ± SD. ^∗^*P* < 0.05, ^∗∗^*P* < 0.01, and ^∗∗∗^*P* < 0.001.

In view of the imbalance of proliferation/apoptosis in PAH, for understanding the role of TUFM in this pathological conversion, relative proteins of apoptosis activator or promotor as well as antiapoptosis factors were examined. The results showed a lower level of antiapoptosis factor Bcl2 and increased expressions of apoptosis activator Bax in the condition of TUFM silence compared with the Si-NC group. However, the overexpression of TUFM cut down the expressions of the apoptosis activator Bax and raised the antiapoptosis factor Bcl2, which resulted in the apoptosis resistance under the hypoxia stimulus (Figures 5(a)–5(d)).

Because the over proliferation of PASMCs in the vascular media was the key pathogenesis of PAH, CCK-8 and EdU tests were used to detect the impact of TUFM on the proliferation of PASMCs. We found that silence of TUFM markedly decreased the cellular proliferation while the overexpression of TUFM facilitated the cell proliferative activity (Figures 5(e)–5(g)).

### 3.9. Alleviated Mitophagy by TUFM Silence Is Associated with AMPK/mTOR Pathway

**Figure 6:**
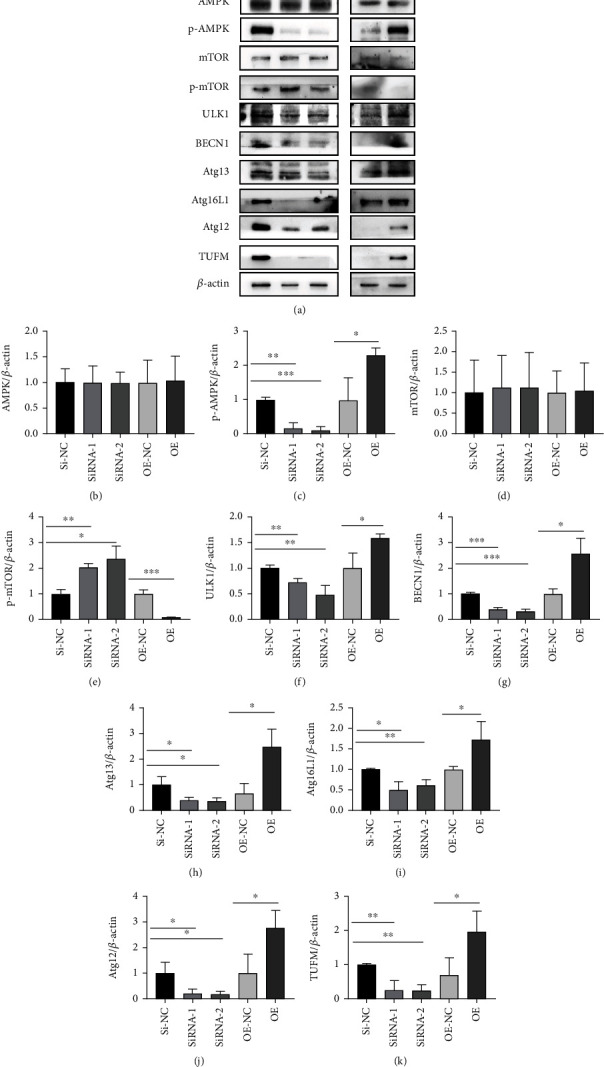
Alleviated mitophagy by TUFM silence is associated with AMPK/mTOR pathway. (a) Representative western blot bands for TUFM, AMPK, p-AMPK, mTOR, p-mTOR, BECN1, Atg13, Atg16L1, ULK1, and Atg12. (b–k) Densitometry data represent the intensity of each group. The data is presented as the mean ± SD. ^∗^*P* < 0.05, ^∗∗^*P* < 0.01, and ^∗∗∗^*P* < 0.001.

In order to gain the underlying mechanism of the TUFM knockdown in mitophagy, we choose AMPK/mTOR pathway, the most well-known signal in the regulation of mitochondrial homeostasis and mitophagy [26] as the downstream factors. Cells treated with Si-TUFM showed a decreased phosphorylated AMPK (p-AMPK), and the overexpression of TUFM led to a high level of p-AMPK compared with the Si-NC group. In contrast, the phosphorylated mTOR (p-mTOR) increased in the condition of TUFM silence and reduced following the overexpression of TUFM. However, the nonphosphorylated style of both AMPK and mTOR is free from the influence of TUFM alternation in PASMCs under the hypoxia circumstance. ULK1 as the main downstream molecule in the mitochondrial biological activity was upregulated by p-AMPK in the OE group, together with increased Atg12, Atg13, and BECN1. The results indicated that the knockdown of TUFM and the following of decreased p-AMPK caused the downregulation of the mitophagy molecules including ULK1, Atg12, Atg13, and BECN1. Similar phenomenon as in the silence of TUFM, the mitophagy factors mentioned above increased along with the overexpressed TUFM and the subsequent p-AMPK augment, while in this signal pathway, the expression level of p-mTOR was contrary to the changes of TUFM and p-AMPK (Figure 6). That is to say, the p-AMPK level has a positive relation to the TUFM variation tendency and p-mTOR has a negative relation. These data revealed that AMPK/mTOR pathway participated in the mitophagy regulated by TUFM in some extent (Figure 6).

## 4. Discussion

**Figure 7:**
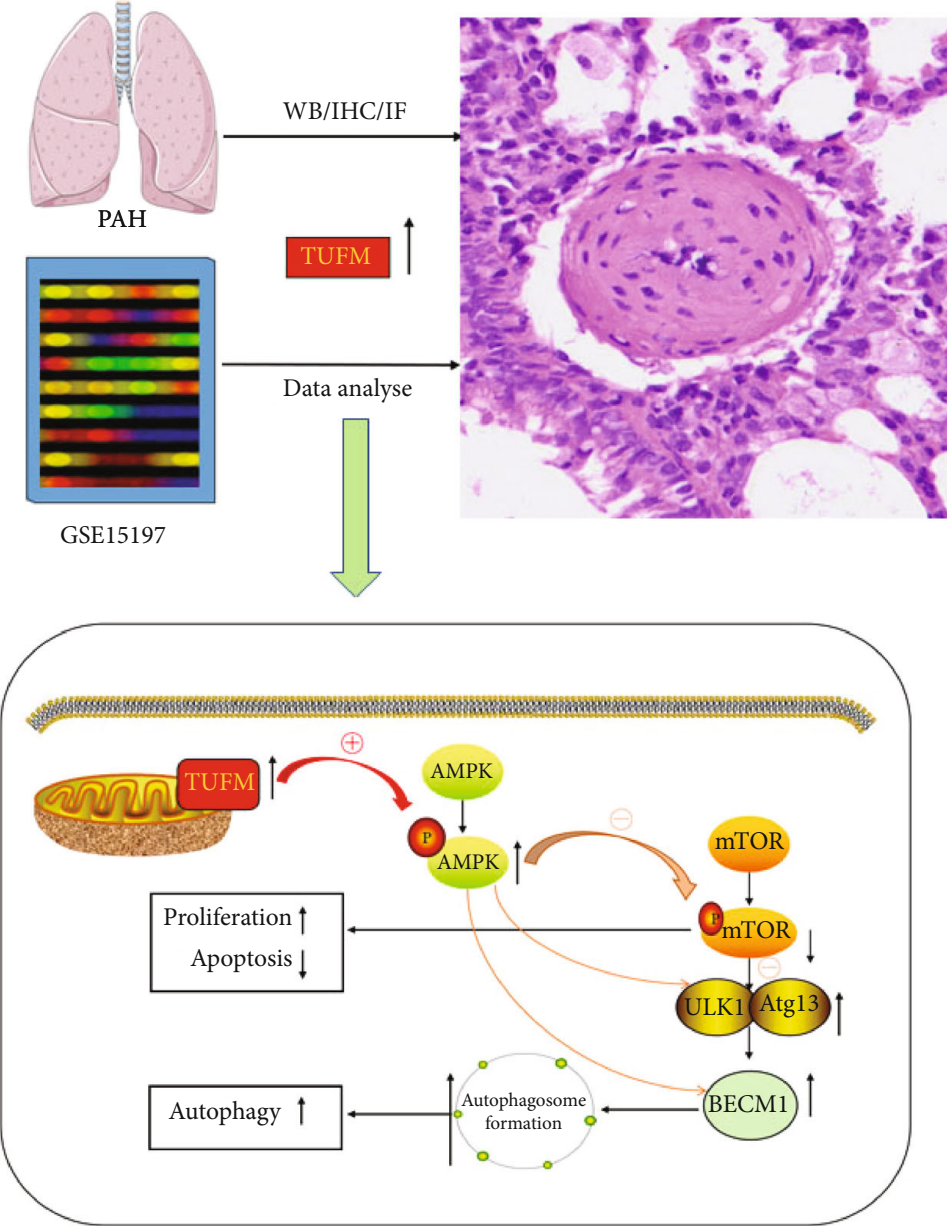
Schematic figure of the current study. Increased TUFM expression in hypoxia-stimulated pulmonary arterial hypertension cells causes an increasing mtDNA translation, leading to dysfunction of the mitochondrial respiratory chain. Mitochondrial dysfunction induces cellular stress and then activates AMPK. On the one hand, activated AMPK decreases the phosphorylation level of mTOR, inhibits the activity of mTOR, and then disassociates from ULK1. Thus, phosphorylation of specific sites of ULK1 and Atg13 is released. Meanwhile, the ULK1 complex is activated through autophosphorylation at thr180 and phosphorylates Atg13, FIP200, atg101, and other Atg proteins. The activated ULK1 complex then translocates to the isolation membrane of the endoplasmic reticulum, where autophagy is initiated. On the other hand, activated AMPK will directly stimulate ULK1 and BECN1, initiating autophagy.

In the current study, we demonstrated that TUFM silence suppresses the development of MCT-induced PAH by regulating mitophagy and correcting apoptosis resistance in PASMCs. The possible mechanism involved AMPK/mTOR pathway which as an important star signal connected apoptosis and mitophagy. This concept (Figure 7) was based on the evidence that (1) GEO data GSE15197 analysis and the previous study on the proteomic analysis [21] suggested the differential expression of TUFM in PAH and normal control; (2) silencing TUFM prevented the rat from development of PAH induced by MCT; (3) Si-TUFM inhibited mitophagy and improved apoptosis of PASMCs under hypoxia condition, and (4) the activity of AMPK and its downstream molecule mTOR were regulated by TUFM in PASMCs.

PAH is a branch of pulmonary hypertension characterized by the remodeling of pulmonary arteriole, leading to the increased pulmonary arterial resistance. The pathogenesis of PAH is complicated and involves heredity, hypoxia, oxidative stress, and unbalanced glycose metabolism, etc. The pathological mainly includes the excessive proliferation and/or apoptosis resistance of PASMCs as well as the accumulation of extracellular matrix components such as collagen and elastin. Mitochondria as the central guardian of mitochondrial homeostasis orchestrate diverse cellular processes including cell cycle progression, mitochondrial dynamics, the cell metabolism, and even the extracellular matrix components [27, 28], which closely connected the pathogenesis and pathology of PAH. TUFM serves as an important factor in the translational expression of mitochondrial DNA and plays a key role in the control of mitochondrial function. Previous study has mentioned that TUFM controls the amino acid elongation of mtDNA-encoded peptides, and the changes in TUFM levels affect the biological activity of various cells [13, 15, 16, 18]. Therefore, combining the pathogenesis of PAH and the relationship between TUFM and mitochondrial function, we assumed that TUFM probably participates the development of PAH. By collecting the data of bioinformatics analysis and related study on proteomic analysis [21], we summarized that differential expression existed between the PAH and normal individuals. At the meanwhile, higher expression of TUFM in MCT-induced PAH rat model was observed compared with normal control rat. In addition, P62, Bcl2, and Bax were tested in the preliminary examination simultaneously to confirm the mitophagy and apoptosis conditions in PAH. As we expected, apart from the increased TUFM level, the reduced P62 was observed in PAH which implies exceeded autophagy. Moreover, high Bcl2 and low Bax expressions were also detected representing the antiapoptosis in PAH. The following immunofluorescent staining demonstrated that TUFM mainly accumulated in the tunica media SMCs of pulmonary arteriole but not the endothelial cells in the inner layer, which were consistent with the major pathological changed cells in PAH.

To prove whether TUFM was involved in PAH development and its role in the pathological process, rats with TUFM silence or overexpression were treated with MCT. Four weeks later, the absence of TUFM inhibits the development of PAH, while the over expression of TUFM aggravates PAH. What is more, the autophagy substrate P62 which has a negative relation with autophagy was at a higher level in the Sh-TUFM group compared with the PAH group, which indicated the decreased mitophagy in absence of TUFM. In addition, autophagy product LC3II/I and BECN1 levels also revealed the diminished mitophagy in the Sh-TUFM group. To our best knowledge, the development of PAH is closely related to the excessive proliferation and apoptosis resistance of PASMC. Reversing the imbalance of proliferation/apoptosis can effectively improve the vascular remodeling and thus decrease the pulmonary arterial pressure. Considering sharp contrast of histological and echocardiography examination between the groups, we thought the effect of TUFM on the apoptosis proteins should be revealed. Interestingly, we found the antiapoptosis Bcl2 was downregulated, and the apoptosis promoting factors Bax and Apaf was increased in the condition of TUFM silence and vice versa. Accordingly, absence of TUFM alleviates the development of PAH in vivo, which might attribute to the mitophagy and apoptosis regulation.

In order to identify the mechanism of the TUFM role in mitophagy and apoptosis regulation during the development of PAH, the in vitro experiment was carried out. As we all know, chronic anoxia induced the proliferation of cells. Even though the hypoxia condition is not absolutely identical to the MCT-induced environment in vivo, it has the same element that stimulates cell proliferation. Consequently, we briefly tested the TUFM expression under different oxygenic conditions and found an increased level of TUFM in response to hypoxia while only slight expression of TUFM in normoxia cells, which was similar to previous study [29]. Cells were treated with TUFM silence or overexpression first and then were exposed in hypoxia to mimic the environment of proliferation. On the one hand, the excessive mitophagy induced by hypoxia was alleviated by Si-TUFM which represented by autophagy markers including the high level of P62 and decreased BECN1 and LC3II/I. On the other hand, hypoxia-induced proliferation of cells was reversed by the absence of TUFM, which was elucidated by the CCK-8 and EdU tests. What is more, Bcl2 was downregulated and Bax was upregulated by knockdown of TUFM, which contributed to the improvement of apoptosis in the circumstance of hypoxia.

To explore the molecular mechanism underlying TUFM knockdown attenuated the mitophagy and proliferation/apoptosis, the well-known signal molecules AMPK and mTOR were focused to illustrate the possible pathway. It is reported that AMPK and mTOR regulate autophagy through the phosphorylation of ULK1, one of the key controlling elements in this process [30]. Therefore, some key markers of mitophagy and the apoptosis indicators together with the active style p-AMPK and p-mTOR were detected for further confirming the role of this pathways in alleviating autophagy induced by TUFM silence in response to hypoxia. Interestingly, the total levels of AMPK and mTOR were not affected by the TUFM levels, but the phosphorylated or activated style of p-AMPK and p-mTOR was regulated by TUFM expression. That is to say, the activity of both AMPK and mTOR was ruled by TUFM in some extent. It is worth noticing that the mitophagy markers ULK1, BECN1, Atg12, Atg13, and Atg16L1 were all under the control of TUFM levels, following by the p-AMPK and p-mTOR regulations. Previous article [31] suggested that TUFM directly combined ULK1 with Atg13 and served as an important regulator for mitophagy and maintained protein homeostasis by initiating the formation of autophagosome. It is suggested that AMPK stimulates ULK1 via phosphorylating its Ser-555 and Ser-777, and mTOR inhibits autophagy by phosphorylation of different Ser residues in ULK1 (Ser-757) [31]. Moreover, the “survival window” of autophagy can be expanded by pretreatment with mTOR inhibitors and/or AMPK activators upon ER stress [31]. These studies indicated the AMPK–mTOR pathway acts as a pivot in the mitophagy and regulates the mitochondrial function and homeostasis. It is lately proposed that mitophagy enhancement in the AMPK–mTOR balance regulates nuclear mRNAs that encode key mitochondrial proteins by controlling the condensation and dissolution of some proteins [32]. However, TUFM is an upstream molecule of AMPK and mTOR, which was confirmed by the evidence of modulation along with the status of knockdown or overexpression. An early study of TUFM (named as EF-Tu) illustrated that TUFM plays a fundamental role in translation conferring charged tRNAs to the ribosomal A-site during peptide chain elongation enabling protein synthesis [33]. In other words, TUFM takes part in the synthesis of mitochondrial proteins by which it influences the mitochondrial function and homeostasis and subsequently modulates the whole cellular function.

As far as the current study is concerned, we conceived that knockdown of TUFM inhibits the development of PAH might be explained as follows: PASMC was stimulated by the outer element, which irritated the proliferation of cells. On the one hand, mitophagy as the most important procedure controlling the mitochondrial functions cleans the fragments and mitochondrial metabolism waste. On the other hand, the clean work done by mitophagy provides the mitochondria with available element to construct the new alternative. What is more, the mitophagy cleans the avenue for the more and more mitochondrial fission that offered the cells more and more energy to complete the proliferation.

However, several limitations of the present study should be mentioned. First, although hypoxia could partially mimic the PAH in vivo environment, it does not totally represent the pathogenesis of MCT-induced PAH. A good in vitro stimulus mimicking PAH for the TUFM study is need. Second, it is a pity that no interference of AMPK or mTOR was used to further confirming the signal pathway. As mitochondrial function regulation is important and complex during the cell cycle process, our next study would focus on both the functional and metabolism regulation of mitochondria to explore the profound mechanism of PAH.

## 5. Conclusion

In this study, we proposed that in vivo TUFM knockdown alleviated PAH induced by MCT and in vitro TUFM silence inhibited mitophagy and improved the proliferation/apoptosis imbalance of PASMCs under hypoxia condition by regulating AMPK/mTOR pathways. Therefore, our study suggested that TUFM may be an essential molecule in mitochondrial autophagy and can at least partially represent a novel targets to inhibit the development of PAH.

## Data Availability

The data that support the findings of this study are available from the corresponding author upon reasonable request.

## References

[1] Bernal-Ramírez J., Silva-Platas C., Jerjes-Sánchez C. (2021). Resveratrol prevents right ventricle dysfunction, calcium mishandling, and energetic failure via Sirt3 stimulation in pulmonary arterial hypertension. *Oxidative Medicine and Cellular Longevity*.

[2] Marshall J. D., Bazan I., Zhang Y., Fares W. H., Lee P. J. (2018). Mitochondrial dysfunction and pulmonary hypertension: cause, effect, or both. *American Journal of Physiology-Lung Cellular and Molecular Physiology*.

[3] Hassoun P. M. (2021). Pulmonary arterial hypertension. *The New England Journal of Medicine*.

[4] Hoffmann R. F., Jonker M. R., Brandenburg S. M. (2019). Mitochondrial dysfunction increases pro-inflammatory cytokine production and impairs repair and corticosteroid responsiveness in lung epithelium. *Scientific Reports*.

[5] Wanderoy S., Hees J. T., Klesse R., Edlich F., Harbauer A. B. (2020). Kill one or kill the many: interplay between mitophagy and apoptosis. *Biological Chemistry*.

[6] Palikaras K., Lionaki E., Tavernarakis N. (2018). Mechanisms of mitophagy in cellular homeostasis, physiology and pathology. *Nature Cell Biology*.

[7] Pickles S., Vigié P., Youle R. J. (2018). Mitophagy and quality control mechanisms in mitochondrial maintenance. *Current Biology*.

[8] Linqing L., Yuhan Q., Erfei L. (2021). Hypoxia-induced Pink1/Parkin-mediated mitophagy promotes pulmonary vascular remodeling. *Biochemical and Biophysical Research Communications*.

[9] Choi C. Y., Vo M. T., Nicholas J., Choi Y. B. (2022). Autophagy-competent mitochondrial translation elongation factor TUFM inhibits caspase-8-mediated apoptosis. *Cell Death & Differentiation*.

[10] Panepinto J. C., Misener A. L., Oliver B. G. (2010). Overexpression of TUF1 restores respiratory growth and fluconazole sensitivity to a Cryptococcus neoformans vad1delta mutant. *Microbiology*.

[11] Lei Y., Wen H., Ting J. P. (2013). The NLR protein, NLRX1, and its partner, TUFM, reduce type I interferon, and enhance autophagy. *Autophagy*.

[12] Lei Y., Kansy B. A., Li J. (2016). EGFR-targeted mAb therapy modulates autophagy in head and neck squamous cell carcinoma through NLRX1-TUFM protein complex. *Oncogene*.

[13] He K., Guo X., Liu Y. (2016). TUFM downregulation induces epithelial-mesenchymal transition and invasion in lung cancer cells via a mechanism involving AMPK-GSK3*β* signaling. *Cellular and Molecular Life Sciences*.

[14] Marcos C. M., Tamer G., de Oliveira H. C. (2019). Down-regulation of TUFM impairs host cell interaction and virulence by Paracoccidioides brasiliensis. *Scientific Reports*.

[15] Kim D., Hwang H. Y., Ji E. S., Kim J. Y., Yoo J. S., Kwon H. J. (2021). Activation of mitochondrial TUFM ameliorates metabolic dysregulation through coordinating autophagy induction. *Communications Biology*.

[16] Xi H. Q., Zhang K. C., Li J. Y., Cui J. X., Zhao P., Chen L. (2017). Expression and clinicopathologic significance of TUFM and P53 for the normal-adenoma-carcinoma sequence in colorectal epithelia. *World Journal of Surgical Oncology*.

[17] Tamai K., Nakamura-Shima M., Shibuya-Takahashi R. (2020). BEX2 suppresses mitochondrial activity and is required for dormant cancer stem cell maintenance in intrahepatic cholangiocarcinoma. *Scientific Reports*.

[18] Weng X., Zheng S., Shui H., Lin G., Zhou Y. (2020). TUFM-knockdown inhibits the migration and proliferation of gastrointestinal stromal tumor cells. *Oncology Letters*.

[19] Kamezaki F., Tasaki H., Yamashita K. (2008). Gene transfer of extracellular superoxide dismutase ameliorates pulmonary hypertension in rats. *American Journal of Respiratory and Critical Care Medicine*.

[20] Ormiston M. L., Slaughter G. R., Deng Y., Stewart D. J., Courtman D. W. (2010). The enzymatic degradation of hyaluronan is associated with disease progression in experimental pulmonary hypertension. *American Journal of Physiology-Lung Cellular and Molecular Physiology*.

[21] Wang J., Uddin M., Li Q. (2022). Identifying potential mitochondrial proteome signatures associated with the pathogenesis of pulmonary arterial hypertension in the rat model. *Oxidative Medicine and Cellular Longevity*.

[22] Li T., Zha L., Luo H. (2019). Galectin-3 mediates endothelial-to-mesenchymal transition in pulmonary arterial hypertension. *Aging and Disease*.

[23] Li T., Li S., Feng Y. (2020). Combination of dichloroacetate and atorvastatin regulates excessive proliferation and oxidative stress in pulmonary arterial hypertension development via P38 signaling. *Oxidative Medicine and Cellular Longevity*.

[24] Liu K., Liu R., Cao G., Sun H., Wang X., Wu S. (2011). Adipose-derived stromal cell autologous transplantation ameliorates pulmonary arterial hypertension induced by shunt flow in rat models. *Stem Cells and Development*.

[25] McMurtry M. S., Bonnet S., Wu X. (2004). Dichloroacetate prevents and reverses pulmonary hypertension by inducing pulmonary artery smooth muscle cell apoptosis. *Circulation Research*.

[26] Herzig S., Shaw R. J. (2018). AMPK: guardian of metabolism and mitochondrial homeostasis. *Nature Reviews Molecular Cell Biology*.

[27] Zachari M., Ganley I. G. (2017). The mammalian ULK1 complex and autophagy initiation. *Essays in Biochemistry*.

[28] Siekacz K., Piotrowski W. J., Iwański M. A., Górski P., Białas A. J. (2021). The role of interaction between mitochondria and the extracellular matrix in the development of idiopathic pulmonary fibrosis. *Oxidative Medicine and Cellular Longevity*.

[29] Ma C., Wang X., He S. (2022). Ubiquitinated AIF is a major mediator of hypoxia-induced mitochondrial dysfunction and pulmonary artery smooth muscle cell proliferation. *Cell & Bioscience*.

[30] Alers S., Löffler A. S., Wesselborg S., Stork B. (2012). Role of AMPK-mTOR-Ulk1/2 in the regulation of autophagy: cross talk, shortcuts, and feedbacks. *Molecular and Cellular Biology*.

[31] Holczer M., Bánhegyi G., Kapuy O. (2016). GADD34 keeps the mTOR pathway inactivated in endoplasmic reticulum stress related autophagy. *PLoS One*.

[32] Fernández-Alvarez A. J., Gabriela Thomas M., Pascual M. L. (2022). Smaug1 membrane-less organelles respond to AMPK and mTOR and affect mitochondrial function. *Journal of Cell Science*.

[33] Clark B. F., Nyborg J. (1997). The ternary complex of EF-Tu and its role in protein biosynthesis. *Current Opinion in Structural Biology*.

